# Methylaluminoxane’s *Molecular Cousins*: Well-Defined Aluminum-Alkyl Borate Salts as Cocatalysts for Olefin Polymerization

**DOI:** 10.1021/acs.accounts.6c00575

**Published:** 2026-09-30

**Authors:** Francesco Zaccaria, Roberta Cipullo, Cristiano Zuccaccia, Christian Ehm, Vincenzo Busico, Alceo Macchioni

**Affiliations:** † Institut de Química Computacional i Catàlisi (IQCC) and Departament de Química, 16738 Universitat de Girona, Girona E-17071, Catalonia, Spain; ‡ Department of Chemical Sciences, Federico II University of Naples, Napoli 80126, Italy; § Department of Chemistry, Biology and Biotechnology, University of Perugia and CIRCC, Perugia 06123, Italy

## Abstract

The success story of molecular olefin polymerization has two protagonists, group 4 metal precatalysts and group 13 cocatalysts, which play equally important roles but have evolved at very different paces. Precatalyst design has shown tremendous progress over the past decades. Conversely, cocatalyst development has almost reached a standstill after methylaluminoxane (MAO) and organic borate salts/tri*-iso*-butylaluminum (TIBAL) binary systems were introduced in the 1980s–1990s. The complexity of innovating cocatalysts arises from the multifaceted roles that they fulfill and the numerous subtleties presiding over their reactivity. Major limitations persist, mostly related to the complex structure of MAO, hampering rational tuning, and to borate salts being “incomplete” activators, since they function only as alkyl abstractors.

Recently, we identified an innovative cocatalyst providing comparable performance to these established activators while combining their distinctive advantages. This system is based on a borate salt of an unusual dinuclear aluminum-alkyl cation, {[*i*Bu_2_(DMA)­Al]_2_(μ-H)}­[B­(C_6_F_5_)_4_] (**AlHAl_DMA**; DMA = *N*,*N*-dimethylaniline), which behaves as a single-component “complete” cocatalyst like MAO, while featuring a well-defined molecular structure like traditional organic borate salts. This species can activate both dialkyl and dichloride precatalysts even in stoichiometric amounts, that is, orders of magnitude lower than those required with MAO. Moreover, it efficiently scavenges impurities, enabling olefin polymerization at catalyst concentrations as low as 10^–7^ M. Its well-defined structure provides opportunities for reactivity modulation.

This Account summarizes the discovery of **AlHAl_DMA**, contextualizing it in the chemistry of olefin polymerization catalysis and organoaluminum compounds. The main features of this novel cocatalyst are discussed by comparing its polymerization performance to those of established cocatalysts. Representative examples also illustrate avenues for polymer microstructure modulation upon substitution of MAO and borate salt/TIBAL with **AlHAl_DMA**. Structural tuning approaches are presented, showing that a novel class of innovative aluminum alkylborate (AAB) salts can be accessed. Some of these novel AAB salts also served as model species to trace the fundamental reactivity of **AlHAl_DMA**. Finally, this Account highlights future challenges in the cocatalyst development for olefin polymerization.

## Graphical abstract

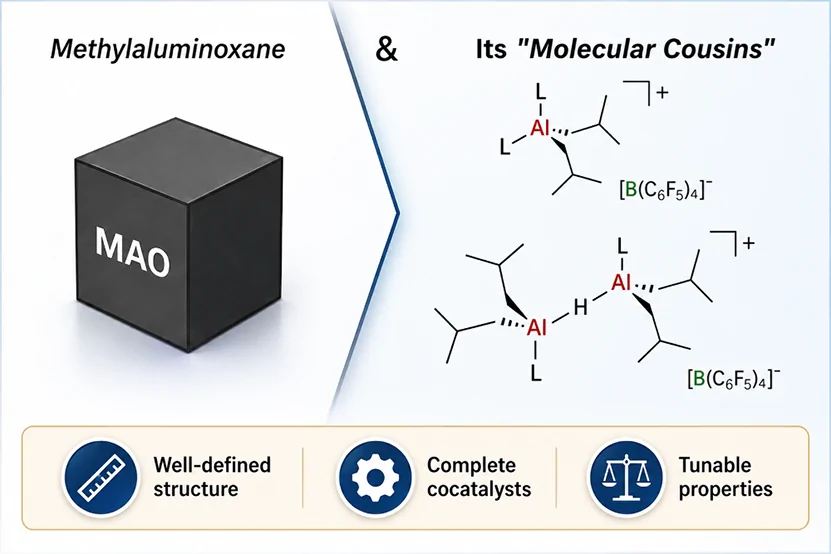

## Key References

-

Zaccaria, F. ; Budzelaar, P. H. M. ; Vittoria, A. ; Macchioni, A. ; Busico, V. ; Ehm, C. Methylaluminoxane’s Molecular Cousin: A Well-defined and “Complete” Al-Activator for Molecular Olefin Polymerization Catalysts. ACS Catal. 2021, 11, 4464–4475 .^1^ A borate salt of an unusual dinuclear Al-alkyl cation (**AlHAl_DMA**) was isolated and found to serve as a complete cocatalyst like methylaluminoxane (MAO).
-

Urciuoli, G. ; Zaccaria, F. ; Zuccaccia, C. ; Cipullo, R. ; Budzelaar, P. H. M. ; Tensi, L. ; Vittoria, A. ; Ehm, C. ; Macchioni, A. ; Busico, V. Al-alkyl Borate Salt Cocatalysts for Olefin Polymerization: Exploration of N-Donor Ligand Variations. Inorg. Chem. Front. 2024, 11, 7872–7885 .^2^ Replacing the dimethylaniline ligand of **AlHAl_DMA** with different N-donor ligands enables reactivity tuning. Preliminary structure–property relationships were identified.
-

Urciuoli, G. ; Vittoria, A. ; Zaccaria, F. ; Zuccaccia, C. ; Cipullo, R. ; Budzelaar, P. H. M. ; Tensi, L. ; Ehm, C. ; Macchioni, A. ; Busico, V. Borate Salts of Aluminum-Alkyl Cations Stabilized by P-, O-, and C-Donors: Synthesis, Characterization, and Application as Cocatalysts. Inorg. Chem. 2025, 64, 9225–9236 10.1021/acs.inorgchem.5c0090640272246.^3^ Replacing the dimethylaniline ligand of **AlHAl_DMA** with different P-, O-, and C-donor ligands enables further reactivity tuning. Rare examples of phosphine or cationic tricoordinate aluminum complexes were reported.
-

Zaccaria, F. ; Urciuoli, G. ; Zuccaccia, C. ; Cipullo, R. ; Budzelaar, P. H. M. ; Menendez Rodriguez, G. ; Tensi, L. ; Vittoria, A. ; Ehm, C. ; Busico, V. ; Macchioni, A. Mechanism of Action of MAO’s Molecular Cousin. ACS Catal. 2025, 15, 17658–17671 10.1021/acscatal.5c05698PMC1260625741234751.^4^ The fundamental reactivity of **AlHAl_DMA** was elucidated at the molecular level with respect to both its activating and impurity scavenging properties.

## 1 Introduction

Long being considered of mere academic interest, molecular catalysts for olefin polymerization have gained a great commercial relevance.^5,6^ They currently represent one-third of polyethylene catalysts market share, and further growth is predicted in view of their relevance for producing advanced materials such as olefin block copolymers^7,8^ and polyolefin elastomers.^9,10^ This success story has two main protagonists: group 4 metal precatalysts^6,11^ and, often overlooked, group 13 cocatalysts.^12^

Group 4 metal catalysts’ capabilities in olefin polymerization have been known since the 1950s.^13,14^ Yet, industrial applications emerged only in the 1990s after the discoveries of *ansa*- and postmetallocene catalysts, exhibiting high selectivity and thermal stability, as well as of methylaluminoxane (MAO) cocatalyst, boosting their productivity by orders of magnitude compared to simpler aluminum-trialkyls.^15,16^ The subsequent tremendous progress in ethylene polymerization has been mostly driven by the discovery of novel precatalyst families, either by serendipity^17–19^ or rational design.^20–27^ Conversely, advances in cocatalysts have been comparatively limited, as the currently most widely used systems were introduced in the 1980–1990s.^12,28,29^

Developing novel cocatalysts is challenging due to the multiple roles they fulfill, namely precatalyst activators,^30^ impurity scavengers,^31^ and -in some cases- chain transfer agents.^32^ Furthermore, the charge of the cationic active species is balanced by a counterion generated by the cocatalyst itself (*vide infra*). In the typical very low polarity solvent used, strong ion pairing interactions between these two ions are present, affecting catalyst activity and selectivity.^29,33–35^ The complex cross-reactivity between precatalysts and cocatalysts may also lead to the formation of dormant or deactivated species.^36–42^

Currently, the two most widely used classes of cocatalysts are MAO (in its various grades)^43,44^ and binary systems consisting of tri-*iso*-butylaluminum (TIBAL) and borate salts like [Ph_3_C]­[B­(C_6_F_5_)_4_] (trityl borate, TTB) or [PhMe_2_NH]­[B­(C_6_F_5_)_4_] (anilinium borate, AB).^12,29,30^ MAO is a “complete” cocatalyst, as it is capable of fulfilling all the key roles demanded to this component: they include activating group 4 metal precatalysts L_n_MX_2_ (L = ancillary ligand, M = transition metal; X = chloride or alkyl), by transforming them into unsaturated cationic alkyl complexes [L_n_M–R]^+^ (R = alkyl), and scavenging impurities to preserve the integrity of the active species. However, despite a solid state structure recently determined by single crystal X-ray diffraction,^45^ the structure of MAO remains strongly debated and shrouded in mystery,^43,46–49^ hindering its rational optimization. In contrast, borate salts activators are structurally well-defined but function only as alkyl abstractors, requiring excess Al-trialkyl as an additional cocatalyst component (*vide infra*). The intrinsic and complementary limitations of these two classes remain unresolved, despite the ever-growing interest for novel cocatalysts.^50–61^

These established cocatalysts share similar activation mechanisms, having a transient aluminum-dialkyl cation as the key intermediate. One of the generally accepted mechanisms for MAO involves formation of AlMe_2_^+^ (Scheme 1a).^43,62–68^ Likewise, it has been demonstrated that the fastest reaction among dichloride precatalyst, TIBAL and borate salts is the one involving the latter two reagents, which generates Al*i*Bu_2_^+^ cations (Scheme 1b): alkylation of the precatalyst by TIBAL is relevant only when these two components are premixed in the absence of borate salts, while no reaction occurs between dichloride complexes and TTB or AB as the latter only serve as alkyl abstractors (*vide supra*).^69–71^ The highly electrophilic AlR_2_^+^ cations react with dichloride precatalyst through chloride abstraction, forming a cationic and coordinately unsaturated [L_n_M–Cl]^+^ species with MAO^–^ or B­(C_6_F_5_)_4_^–^ counterions. Subsequent rapid alkylation by excess aluminum-alkyl generates the [L_n_M–R]^+^ active species (Scheme 1a,b).

**Scheme 1:**
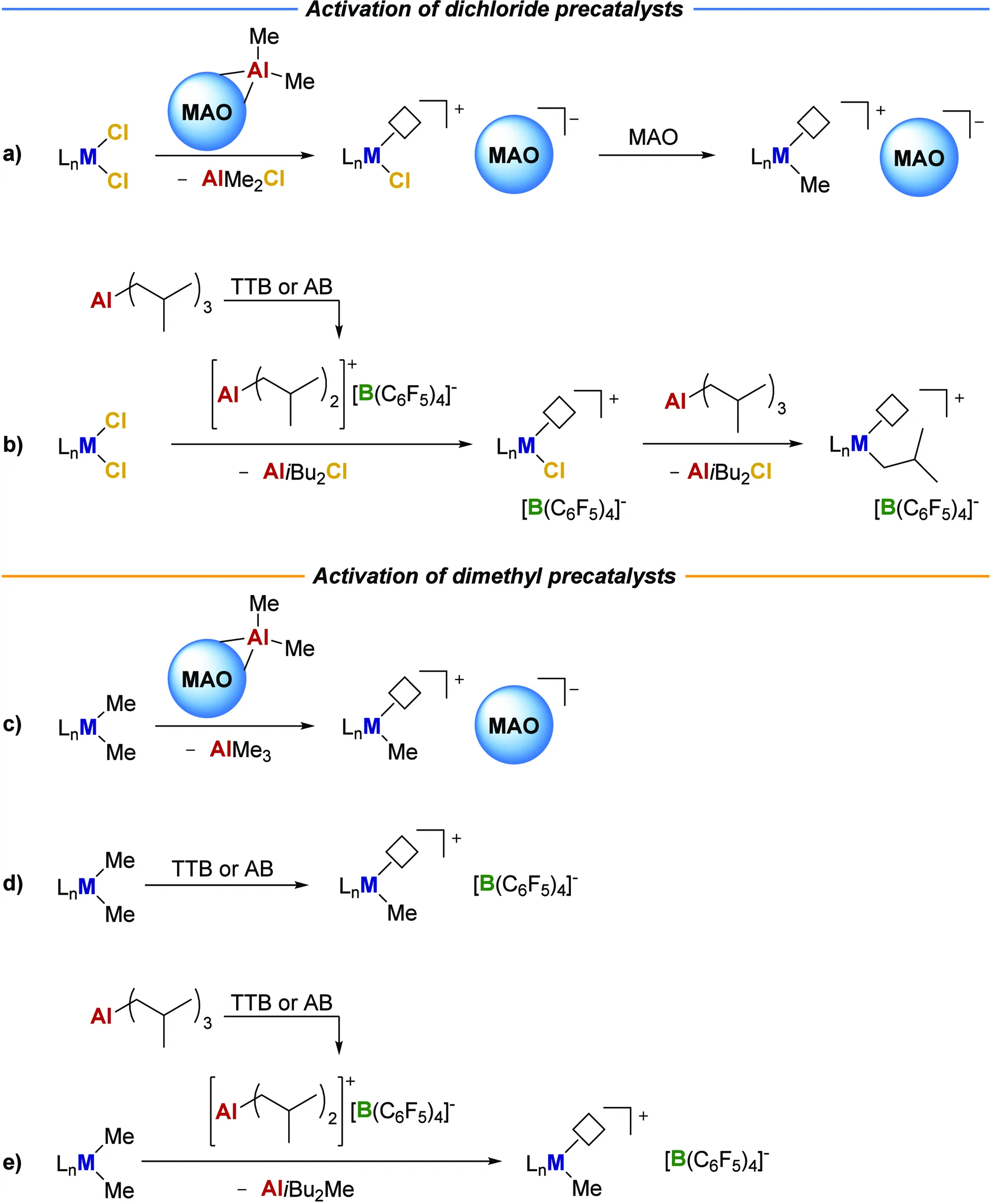
Activation of Dichloride Precatalysts by (a) MAO via AlMe_2_^+^ or (b) Borate Salts/TIBAL via Al*i*Bu_2_^+^ and of Dimethyl Precatalysts by (c) MAO via AlMe_2_^+^ or Borate Salts (d) in the Absence or (e) Presence of TIBAL^a^

a M = group 4 metal; L = ancillary ligand; TTB = [Ph_3_C]­[B­(C_6_F_5_)_4_]; AB = [PhMe_2_NH]­[B­(C_6_F_5_)_4_].

In the case of dialkyl precatalysts, activation by MAO follows a similar path (Scheme 1c), while direct alkyl abstraction between borate salts and precatalysts becomes possible (Scheme 1d). The latter simplified catalytic system, not requiring TIBAL, has been deeply exploited in fundamental investigations of reaction mechanisms in olefin polymerization.^12,29,30,33^ Nevertheless, in real life polymerization experiments, an impurity scavenger like TIBAL is always required and the dominant activation pathway again involves Al*i*Bu_2_^+^ (Scheme 1e): the latter observation has been demonstrated, for instance, in the reaction between dimethyl precatalysts, TIBAL and TTB that selectively leads to Ph_3_CH and isobutene, deriving from hydride abstraction from TIBAL by Ph_3_C^+^, and not Ph_3_CMe, expected from the direct reaction between L_n_MMe_2_ and TTB.^69,71^

During a recent study on highly active metallocene catalysts, we observed an unexpected difference in activation kinetics with TTB/TIBAL vs AB/TIBAL: the same maximum activity could be achieved, consistent with the fact that they both operate via the same Al*i*Bu_2_^+^ cation (Scheme 1b), but an induction delay was observed with AB.^72^ This prompted a reinvestigation of the borate salts/TIBAL activation chemistry that, quite serendipitously, led to the discovery of a novel class of cocatalysts. The new species combine the distinctive advantages of established systems, behaving as “MAO’s molecular cousins”. This *Account* discusses the history and properties of these compounds, highlighting their peculiarity in organoaluminum chemistry and their relevance in olefin polymerization catalysis.

## 2 The Discovery of the Prototype: **AlHAl_DMA**

The main difference between the reactions of TIBAL with TTB and AB lies in their byproducts. The Ph_3_C^+^ cation of TTB abstracts a hydride from an *iso*-butyl group of TIBAL forming, along with [Al*i*Bu_2_]­[B­(C_6_F_5_)] (Scheme 1b), triphenylmethane and *iso*-butene.^69,71^ Conversely, the Brønsted acidic PhMe_2_NH^+^ in AB hydrolyzes an Al–*i*Bu bond leading to *iso*-butane and *N*,*N*-dimethylaniline (**DMA**).^70^ All these byproducts should be inert under polymerization conditions; even the aniline is known not to interact with sterically hindered activated metallocenes.^73^ Nevertheless, a Lewis base like **DMA** can hardly remain isolated in the presence of the numerous Lewis acids in the catalytic pool: this donor molecule should be trapped, if not by the catalyst, at least by the scavenger under typical polymerization conditions. We envisioned that the interaction between **DMA** and TIBAL could be responsible for the generation of tamed Al*i*Bu_2_^+^ cations, explaining the above-mentioned differences between AB/TIBAL and TTB/TIBAL.^72^

Our search for donor stabilized Al*i*Bu_2_^+^ eventually led to two compounds, first detected as coproducts in the reaction of TIBAL, **DMA** and substoichiometric amounts of TTB (≤0.3 equiv; Scheme 2a). They correspond to borate salts of a simple bis-amine Al–*i*Bu cation, [Al*i*Bu_2_(DMA)_2_]^+^ (**Al_DMA**), and a rather unusual dinuclear cation with two aluminum centers binding two *iso*-butyl groups, one **DMA** and a bridging hydride, {[*i*Bu_2_(DMA)­Al]_2_(μ-H)}^+^ (**AlHAl_DMA**; Figure 1). Conclusive evidence for their structural assignment was obtained by combining mass spectrometry (*m*/*z* 383 for **Al_DMA** and *m*/*z* 525 for **AlHAl_DMA**), IR spectroscopy (Al–H–Al stretching at 1604 cm^–1^) and detailed 2D NMR characterization, particularly ^1^H NOESY NMR that evidenced dipolar correlations among Al–*i*Bu, Al–DMA, and, in the case of the dinuclear product, the bridging hydride at δ­(^1^H) = 2.86 ppm (Figure 1).^1^

**Scheme 2:**
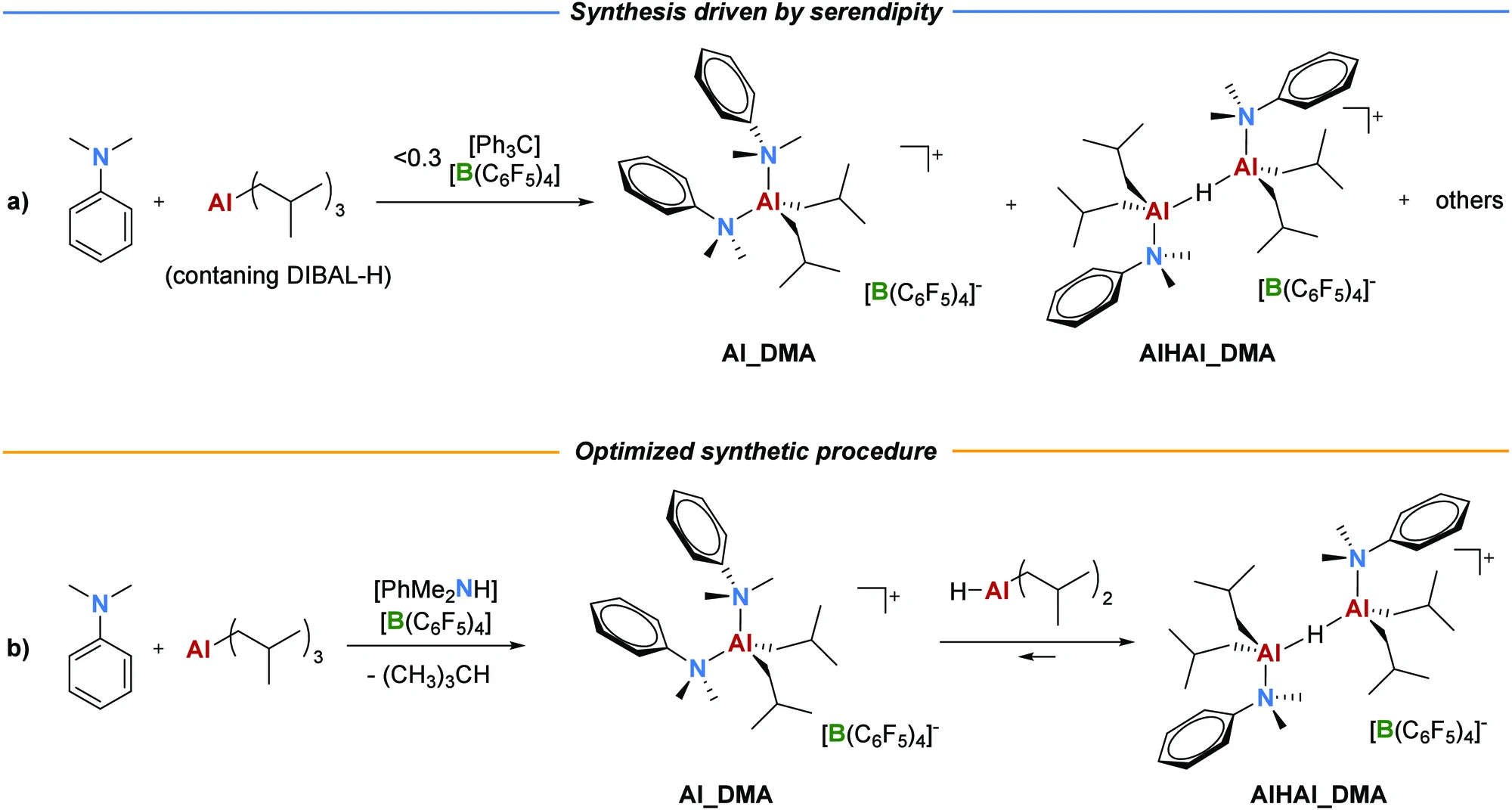
Serendipitous (a) and Optimized (b) Synthesis of AlHAl_DMA and Al_DMA; DIBAL-H Is Shown as a Monomer for Simplicity

**Figure 1:**
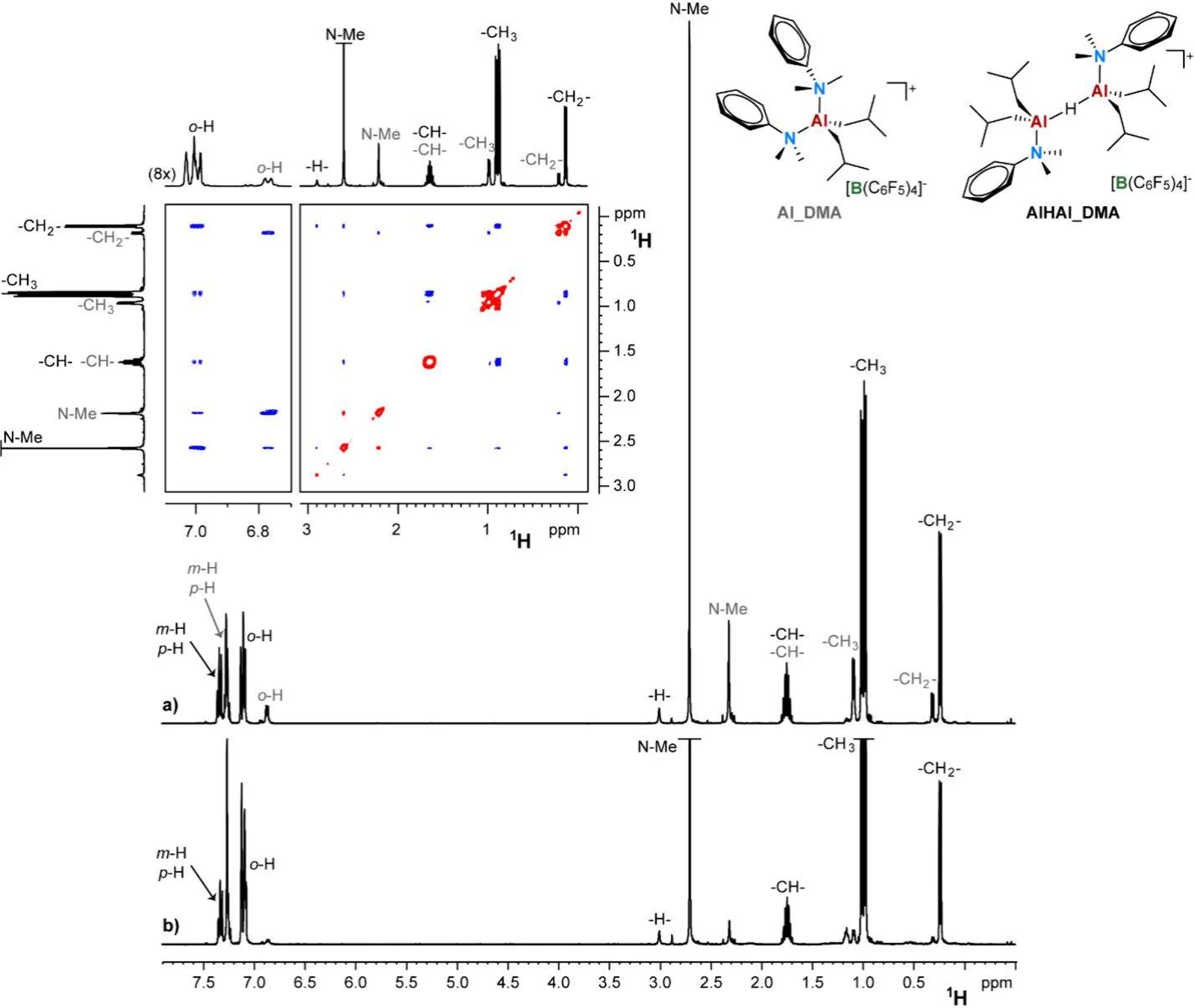
^1^H NMR spectra (chlorobenzene-*d*_5_, 298 K) of (a) a mixture of **AlHAl_DMA** and **Al_DMA** derived from the reaction in Scheme 2a (inset: corresponding ^1^H-NOESY NMR spectrum) and (b) enriched solution in **AlHAl_DMA** obtained as in Scheme 2b. Adapted with permission from ref 1. Copyright 2021 American Chemical Society.

The hydride in **AlHAl_DMA** originates from di-*iso*-butylaluminum (DIBAL-H), which is a ubiquitous impurity of TIBAL deriving from its self-decomposition. In fact, the optimized synthetic protocol for the new Al-alkyl borates salts consists in reaction of equimolar amounts of TIBAL, **DMA**, and AB to obtain **Al_DMA**, which is then converted into **AlHAl_DMA** by addition of 1 equv of DIBAL-H (Scheme 2b). The mono- and dinuclear species exist in equilibrium, as further discussed in the following sections.^1^ It is worth noting that such a dynamic nature is reminiscent of that of proposed among the different components of MAO,^43,49^ with the crucial difference that in this case the various components are structurally well-defined and fully characterizable, providing key advantages for rationalization.

While analogs of **Al_DMA** are known,^74^ **AlHAl_DMA** is a very peculiar compound. In difference to common dibridged Al–(μ-H)_2_–Al species, singly bridged Al–(μ-H)–Al motifs are rare and occasionally found in dialuminum anions (e.g., Na­[(Me_3_Al)_2_(μ-H)]; Figure 2a)^75^ or neutral complexes featuring anionic κ^2^-ligands (Figure 2b,c).^76,77^ **AlHAl_DMA** is indeed the only example of cationic complex containing a singly bridged Al–(μ-H)–Al motif. Unfortunately, it is a hardly crystallizable oil at room temperature, so far hampering solid-state structural analysis by X-ray diffraction. Its DFT optimized 3D-structure features a *C*_2_-symmetric geometry with identical Al–H bonds (1.778 Å) and nearly linear Al–H–Al configuration (175°; Figure 3). The charge is equally distributed over the three-center two-electron bond, and the LUMO extends over the Al–H–Al fragment and **DMA**.^1^

**Figure 2:**
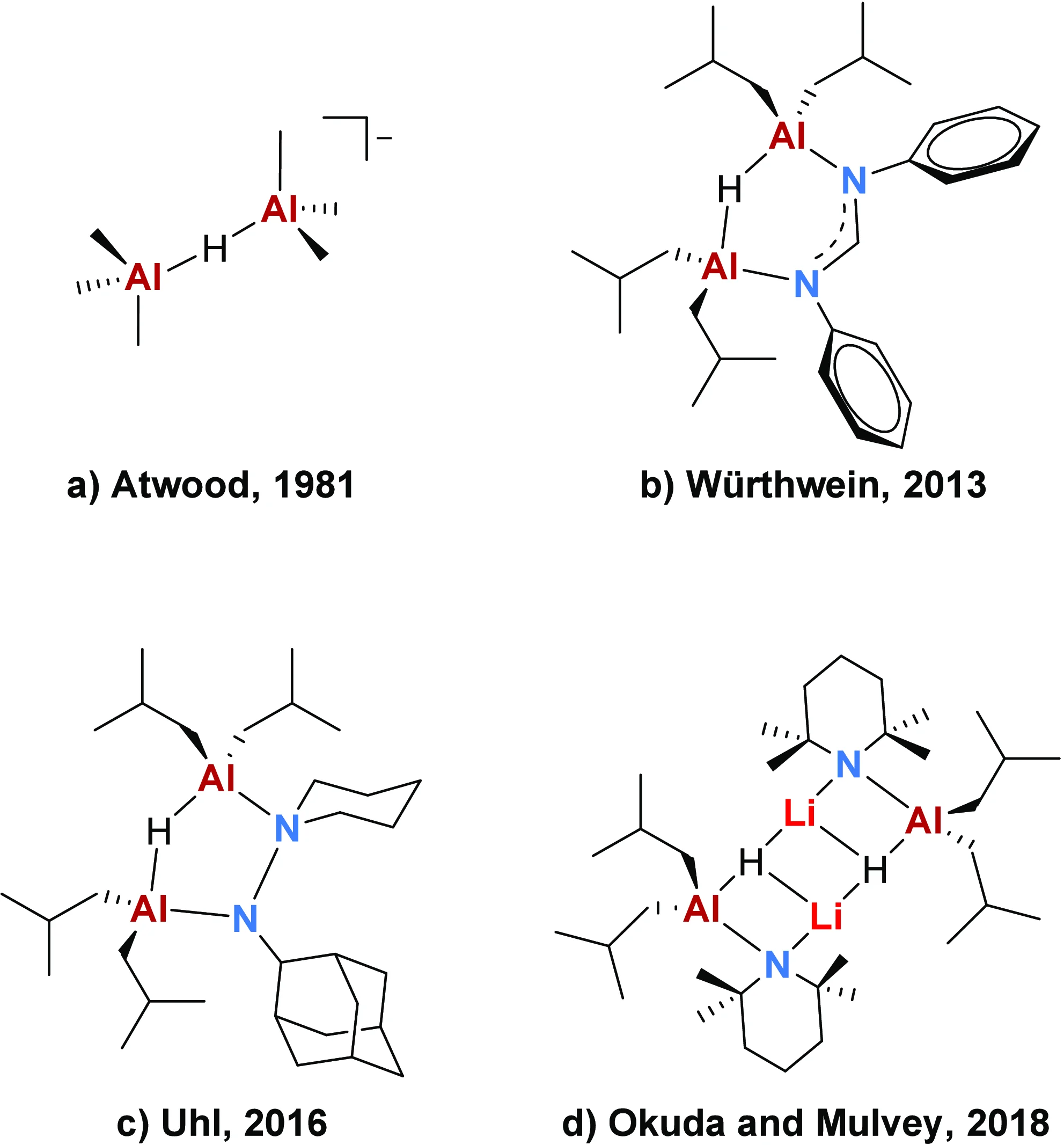
Examples of compounds containing singly bridged Al–(μ-H)–Al motif: (a) dialuminum anions, (b,c) neutral complexes with anionic κ^2^-ligands, and (d) mixed Al/Li hydrides.

**Figure 3:**
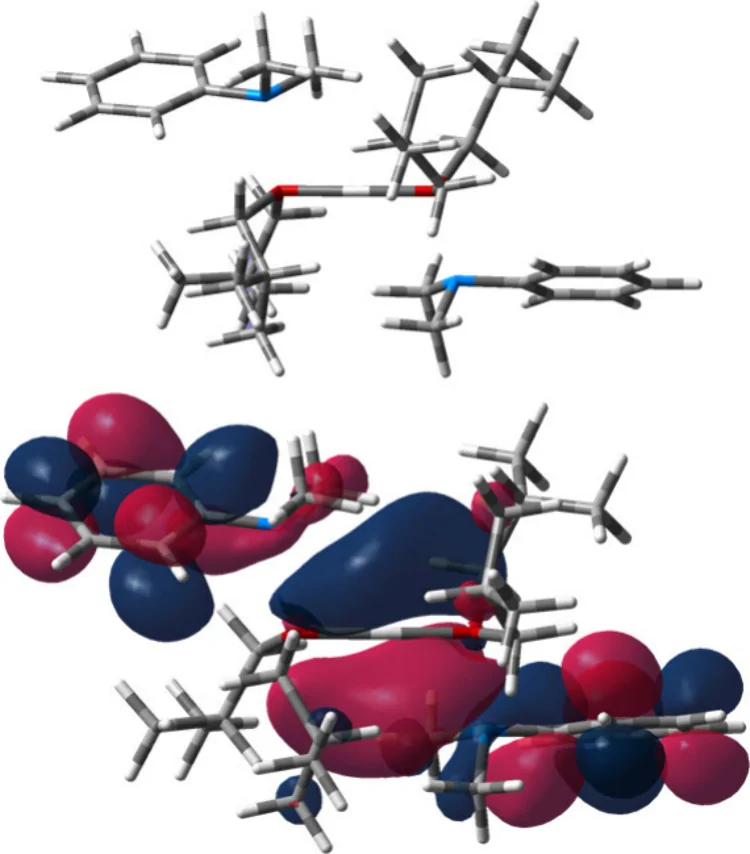
DFT structure of **AlHAl_DMA** (top) and its delocalized LUMO (bottom, isovalue = 0.02). Al in red, N in light blue, C in gray, H in white. Adapted with permission from ref 1. Copyright 2021 American Chemical Society.

Regarding the reactivity of **AlHAl_DMA**, it is worth mentioning another related species that is the mixed Al/Li-hydride reported by Okuda, Mulvey, and co-workers, featuring Al*i*Bu_2_ fragments binding a N-ligand and a bridging hydride, and exhibiting catalytic activity in the hydroboration of carbonyls (Figure 2d).^78^ Considering the surging relevance of Al-hydrides,^79,80^ **AlHAl_DMA** might have still unexplored potentialities in organic synthesis. However, the characteristic that mainly drew our attention and fostered further investigation was that **AlHAl_DMA** resembles a tamed Al*i*Bu_2_^+^ cation of the type hypothesized in the activation chemistry of AB/TIBAL. In fact, we could demonstrate that this species forms also as an initial transient species in the reaction between TIBAL and AB.^1^ The methylated analogue of **Al_DMA** was known to have modest activating ability in olefin polymerization,^74^ possibly because coordination of two **DMA** ligands saturates and suppresses the Lewis acidic character of the metal center. Conversely, **AlHAl_DMA** can be described as Al*i*Bu_2_^+^ stabilized by a single **DMA** unit and a more weakly binding Al*i*Bu_2_(**DMA**)H fragment (Figure 1), making it a more promising candidate for applications as an activator in olefin polymerization.

## 3 Polymerization Performance: Exploring Precatalyst-Cocatalyst Combinations

Testing of **AlHAl_DMA** with a variety of group 4 metal precatalysts (Figure 4) revealed several interesting features of this compound. Already from the first polymerization experiments, it emerged that **AlHAl_DMA** is not only an effective activator but a “complete” cocatalyst. In combination with **Cat-1** (Figure 4), no activity is observed with mononuclear **Al_DMA** unless TIBAL is added, while dinuclear **AlHAl_DMA** functions as a standalone cocatalyst like MAO (Table 1, entries 1 vs 2–3). One equivalent of this novel aluminum alkyl borate (AAB) salt can activate **Cat-1** in propene polymerization at moderately low catalyst concentration (10^–5^ M; entry 2); at industrially relevant concentrations (10^–7^ M; entry 3), just 50 equiv. suffice. Notably, comparable productivity to MAO is obtained at substantially lower Al/Zr ratios (10^2^ vs 10^4^; entries 3–4), demonstrating that **AlHAl_DMA** is not only competitive with the best-in-class cocatalyst but also offers an advantage in terms of reduced Al/M ratios necessary for optimal performance (M = group 4 metal).^1^ Considering its reactivity and well-defined structure, it therefore deserves the title “MAOs’ molecular cousin”.^1^

**Figure 4:**
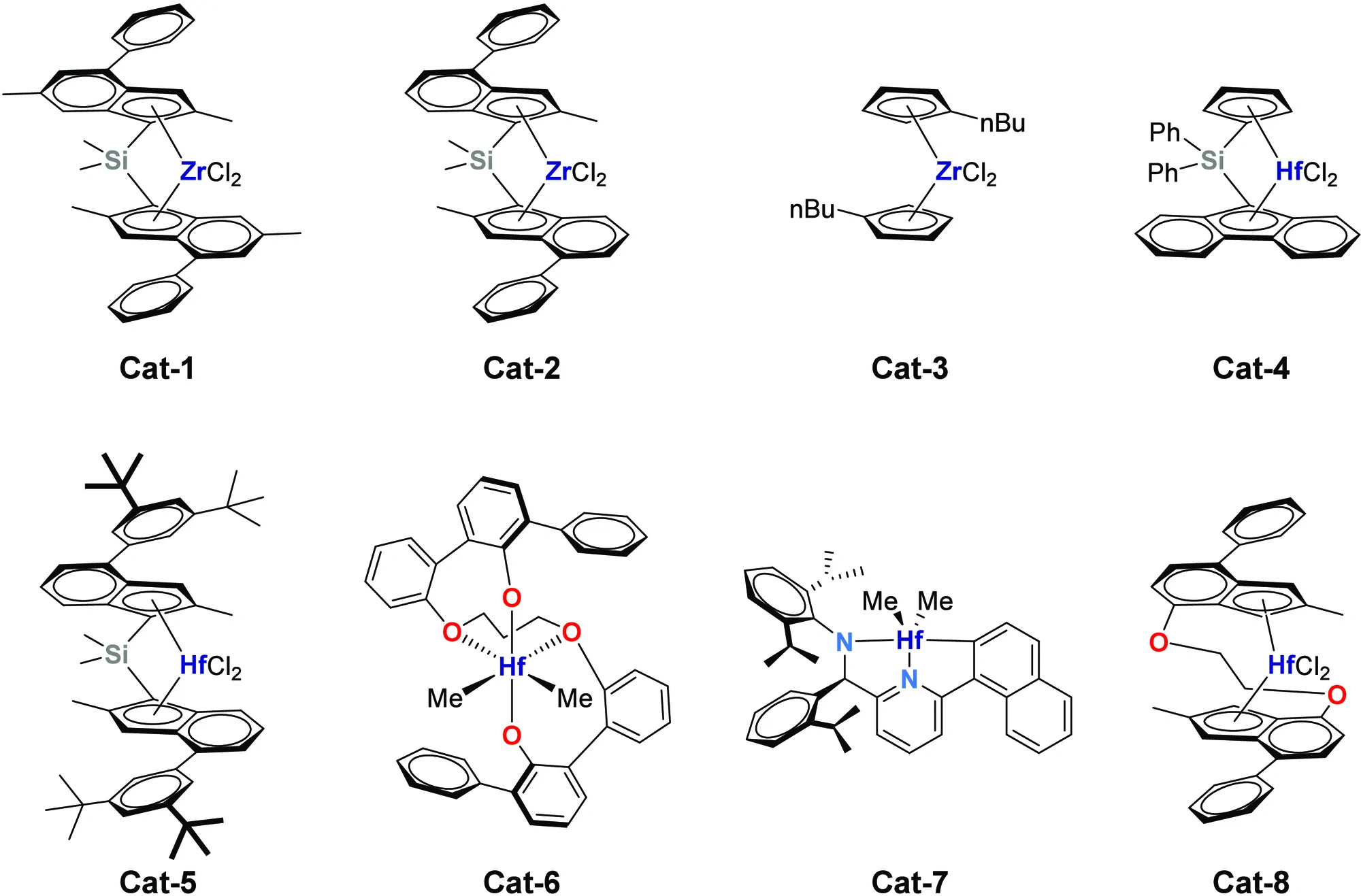
Catalysts tested in combination with **AlHAl_DMA**.

**Table 1:** Selection of Reported Polymerization Experiments Comparing the Performance of **AlHAl_DMA** with Those of Established Cocatalysts^a^

entry	cat.	*T* (°C)	*p* (bar)	olefin	cocatalyst	[Al]/[M]^b^	[B]/[M]	[M] (10^6^ M)	*R*_p_ ^c^	*M*_n_ (kDa)	PDI
1	**1**	60	6.6	propene	**Al_DMA**	100	50	2.0	∼0	n.a.	n.a.
2^d^					**AlHAl_DMA**	2	1	25	≫0	n.a.	n.a.
3					**AlHAl_DMA**	100	50	0.4	185	300	2.2
4					MAO	15000		0.2	169	360	2.6
5^e^					TTB/TIBAL	5000–2500	10	0.2–0.4	182–1592	380	2.3
6					AB/TIBAL	400	2	2.5	32	390	2.0

7	**2**	60	4.5	eth/hex	**AlHAl_DMA**	100	50	0.3	268	92	2.4
8					**AlHAl_DMA**	133	67	0.3	313	80	2.5
9					**AlHAl_DMA**	200	100	0.3	462	80	2.3
10					**AlHAl_DMA**	333	167	0.3	485	82	2.3
11					**AlHAl_DMA**	433	217	0.3	469	85	2.4
12^d^					**AlHAl_DMA**	60	30	0.5	≫0	n.a.	n.a.
13					AB/TIBAL	3333	2	0.3	∼0	n.a.	n.a.
14					AB/TIBAL	500	2	2.0	124	87	2.5

15	**3**	100		1-octene	**AlHAl_DMA**	10	5	10	2.6	0.60	^f^
16					MAO	2000		10	12	0.39	^f^
17					AB/TIBAL	200	2	10	8.9	0.54	^f^
18					TTB/TIBAL	200	2	10	13	0.57	^f^
19					MMAO-12	2000		10	14	0.42	^f^
20					MAO/BHT	2000		10	2.7	0.43	^f^

21	**4**	60	2.0	propene	**AlHAl_DMA**	10	5	20	5.5	340	2.3
22					MAO	500		20	0.11	54	2.0
23					MAO/BHT	500		20	1.3	399	2.2
24					TTB/TIBAL	50	5	20	1.6	411	2.4

25	**5**	60	2.0	propene	**AlHAl_DMA**	20	10	12	3.2	52	2.2
26					MAO	333		30	0.05	91	2.9
27					MAO/BHT	333		30	0.12	136	2.9
28					TTB/TIBAL	100	5	12	3.3	74	2.3

29	**6**	60	2.0	propene	**AlHAl_DMA**	20	10	10	3.1	55	2.3
30					MAO	1000		10	3.6	5.6	3.8
31					MAO/BHT	1000		10	8.4	299	2.3
32					TTB/TIBAL	100	1	10	4	31	2.2

33	**7**	60	2.0	propene	**AlHAl_DMA**	50	25	4.0	5.9	296	3.2
34					MAO	750		4.0	4	11	6.1
35					MAO/BHT	750		4.0	27	801	2.4
36					MAO/BHT	2500		4.0	24	678	2.4
37					TTB/TIBAL	250	2	4.0	24	60	2.9

38	**8**	30	4.0^g^	ethene	**AlHAl_DMA**	60	30	34	0.4	1.0	2.7
39					TTB/TIBAL	60	5	34	0.16	5.9	3.4

a All experiments were performed in toluene, except entries 15–20 that were performed in neat monomer.

b [Al] = total concentration of aluminum, as derived from MAO, TIBAL, or **AlHAl_DMA**.

c Productivity in kg_PE_ • mmol_Cat_^-1^ • h^–1^.

d High productivity but uncontrolled kinetic regimes.

e Poor reproducibility, see main text.

f PDI not reported, see main text and ref 81.

g Including pressure of inert gas, see ref 82 Eth/Hex = ethene/1-hexene copolymerization at [H]_feed_ = 1.8 v/v %. Data were taken from refs 1 (entries 1–6),^2^ (entries 7–14),^81^ (entries 15–20),^83^ (entries 21–37), and^82^ (entries 38–39). n.a. = not available

Compared to classical borate salts, **AlHAl_DMA** delivers similar productivities to TTB/TIBAL, while ensuring better kinetic control of polymerization (Table 1, entry 3 vs 5; for a detailed discussion on polymerization kinetics with TTB/TIBAL, see ref 72). Furthermore, it outperforms AB/TIBAL, enabling propene (entry 3 vs 6) and ethene/1-hexene polymerization (entry 7 vs 13–14) even at low catalyst concentrations, where no activity is observed with the established cocatalyst.^1,2^ This highlights the strong scavenging ability of **AlHAl_DMA**. Systematic exploration revealed that [**AlHAl_DMA**] as low as 50 μM suffices to achieve optimal scavenging and activation of **Cat-2** at [Zr] = 3 × 10^–7^ M in ethene/1-hexene copolymerization (Table 1, entries 7–11).^2^

Effective scavenging at such low cocatalyst concentration was achieved also in 1-octene oligomerization experiments with the industrially relevant **Cat-3** (Figure 4).^81^ The productivity obtained with **AlHAl_DMA** is lower compared to borate salts/TIBAL and various MAOs grades (Table 1, entries 15–20). Yet, it is quite satisfying considering that it was achieved at very low [B] = 50 μM and [B]/[Zr] = 5, and that higher impurity concentrations are expected when working in neat liquid monomer compared to solutions of gaseous monomers. Furthermore, a well-defined Schultz–Flory product distribution was obtained with this novel AAB salt, comparably with TTB/TIBAL but in contrast to the bimodal distributions obtained with MAOs.^81^ Considering the importance of selectivity in α-olefins oligomerization for the industrial production of lubricants,^84^ these results further emphasizes the advantage of working with a molecularly well-defined MAO’s molecular cousin.

Indeed, analyzing cocatalytic properties includes evaluation of possible side reactions with the cationic active species that might affect activity as well as selectivity. Hafnium-based precatalysts are particularly suitable probes, due to their tendency to form stable heterodinuclear adducts with Al-alkyls, particularly residual trimethylaluminum (TMA) in MAO: these adducts can lead to catalyst deactivation and/or chain termination.^36–42,85–87^

A first study on **AlHAl_DMA** in combination with hafnium catalysts reported a systematic comparison in propene polymerization with TTB/TIBAL, MAO and a modified MAO, obtained by adding hindered phenol to scavenge TMA (MAO/BHT;^88,89^ BHT = 2,6-di-*tert*-butyl-4-methylphenol).^83^ The new cocatalyst provides the best performance in terms of productivity and polymer molecular weight (MW) with both *C*_s_ and *C*_2_-symmetric *ansa*-metallocenes (**Cat-4** and **Cat-5**; Figure 4), comparably to TTB/TIBAL (Table 1, entries 21–28). Even though **AlHAl_DMA** cannot match MAO/BHT’s performance with postmetallocenes **Cat-6** and **Cat-7** (Figure 4), it leads to productivity and polymer MW in the same order of magnitude (Table 1, entries 29–37).^83^ The results obtained with **Cat-7** are particularly significant, since the open active pocket and reactive Hf-aryl bond of this catalyst make it particularly susceptible to side-reactions with the cocatalyst.^7,17,90–93^ Irreversible transalkylation is typically the preferred chain termination route even with bulky Al-alkyls like TIBAL.^7,92–97^ The high polymer MW obtained with **AlHAl_DMA** demonstrates the low tendency of this novel cocatalyst to trigger chain transfer to aluminum.^83^

Cocatalyst effects on polymer microstructure were small or negligible for the above-mentioned Hf catalysts.^83^ Conversely, a marked effect was observed by the group of Rieger in ethylene homopolymerization by a unconventional dialkoxy-bridged *ansa*-hafnocene (**Cat-8**; Figure 4), producing polyethylene containing exclusively methyl branches.^82^ This feature is unusual, since methyl side chains typically form along with longer ramifications. **Cat-8** in combination with **AlHAl_DMA** delivered a 2.5 times higher productivity compared to TTB/TIBAL (Table 1, entries 38–39). This is accompanied by a decrease in polymer MW and methyl branch density, dropping from 66‰ to 43‰. The authors traced this decrease to a suppression of β-H elimination during polymerization, in line with this process being generally accepted as a key step in chain branching formation.^98,99^ However, it remains unclear how cocatalyst variation might trigger such suppression: ion pairing effects can be excluded since **AlHAl_DMA** and TTB provide the catalyst with the same perfluorophenyl borate anion.^29,33^ More likely, such a marked effect can be ascribed to an interaction of the AlHAl-cation, or derivatives thereof, with the transition metal active species. Also in this case, the role of a Lewis base as DMA might be crucial.^100^ This exemplifies the complexity of as well as the opportunities in controlling precatalyst/cocatalyst cross reactivity, as further discussed in the following sections.

## 4 Polymerization Performance: Exploring Structural Variations of the Cocatalyst

**AlHAl_DMA** offers various handles for reactivity tuning by molecular design, namely, the nature of the Al-alkyl group, neutral ligand (**L**), bridging group, and anion. Variation of **L** is the simplest and most investigated approach, which enabled isolation of nearly 30 **Al_L** and/or **AlHAl_L** variations bearing different N-, P-, O-, and C-donor ligands (Scheme 3). These species can be prepared as the prototypes of this class by reaction of TIBAL, **L**, and borate salt of protonated **L** (Scheme 3a). If the latter is not readily available, two equivalents of free ligand and TIBAL can be reacted with TTB or, for **L** = strong Lewis basis, AB (Scheme 3b,c). Although these alternative routes have worse atom economy, they have been conveniently exploited for rapid ligand screening.^2,3,101^

**Scheme 3:**
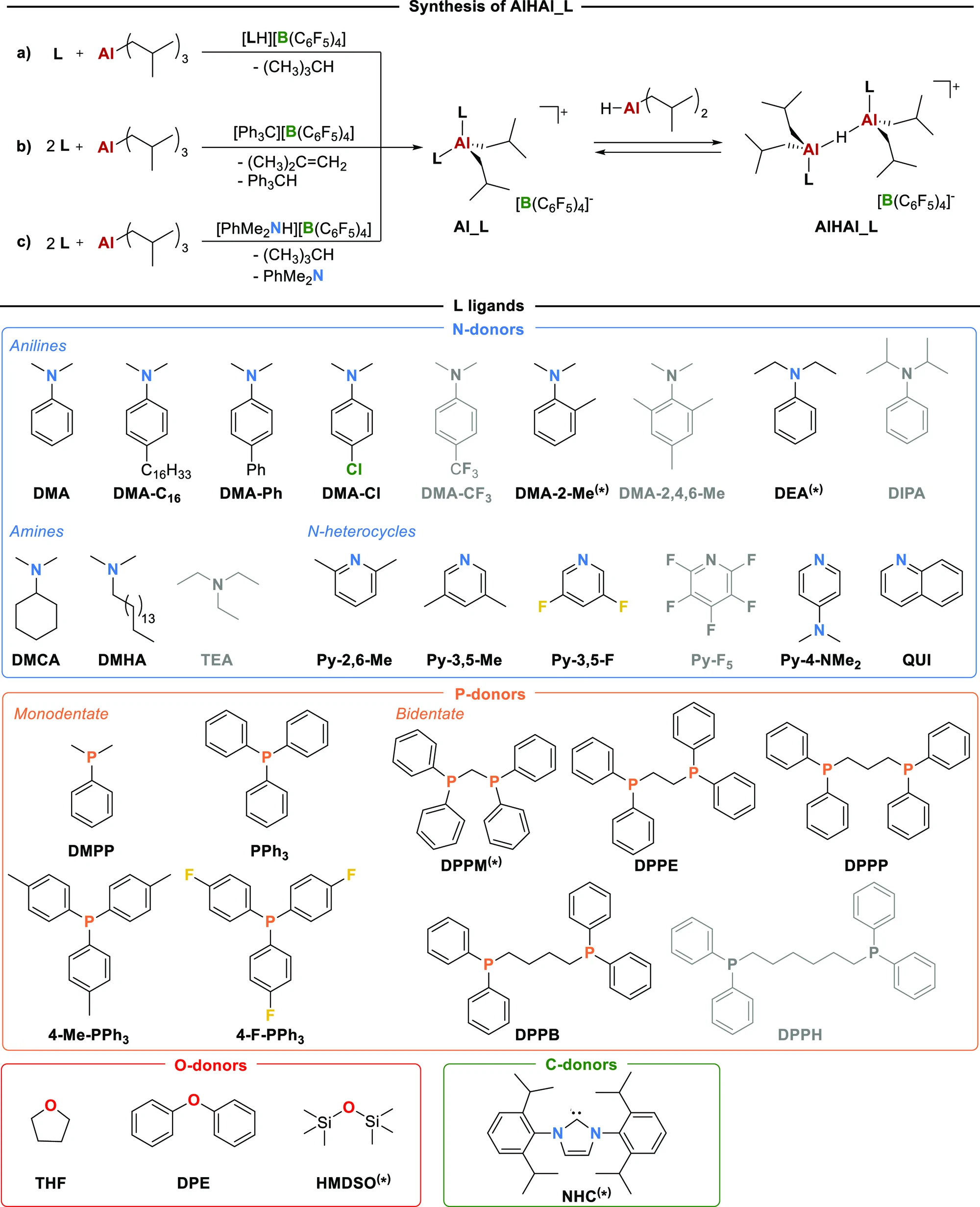
Synthesis of AlHAl_L Cocatalysts *via* (a) Optimized Reaction with Borate Salts of L-H^+^ or Using (b) TTB and (c) AB as Ionizing Agents, and L Ligands Explored^a^

a DIBAL-H is shown as a monomer for simplicity. Species giving a complex mixture of decomposition products are reported in grey. ^(^*^)^ = ligands selectively giving unexpected yet stable products.

Only few unsuccessful synthetic attempts were reported among the donors studied (gray structures in Scheme 3).^2,3^ They include **DMA-2,4,6-Me**, **DIPA**, and **TEA**, which are too bulky to effectively coordinate to Al, as well as **DMA-CF**_**3**_ and **Py-F**_**5**_, which undergo C–F bond activation. Interestingly, stable **Al_L** could be obtained with **Py-3,5-F**, likely due to the presence of less activated C–F bonds compared with perfluorinated **Py-F**_**5**_, evidencing the complex trade-off between preserving and controlling the strong Lewis acidity of these species.

Moreover, a few **L** provided unexpected yet stable products.^2,3^ They include three rare examples of tricoordinate Al-alkyl cations with **DEA**, **NHC** or **HMDSO** ligands (Figure 5a), which are too bulky to allow coordination of two **L**, yet capable to bind and shield the highly reactive Al*i*Bu_2_^+^. **DPPM**, in difference to the other diphosphines, coordinates to Al as a monodentate ligand, likely due to its methylene bridge being too short for κ^2^-coordination (Figure 5b). **Al_DMA-2-Me** forms but rapidly decomposes via benzylic C–H activation giving neutral cyclometalated **Al_cyclo1** (Figure 5c), while polynuclear species were obtained with **DPPH**.^2,3^

**Figure 5:**
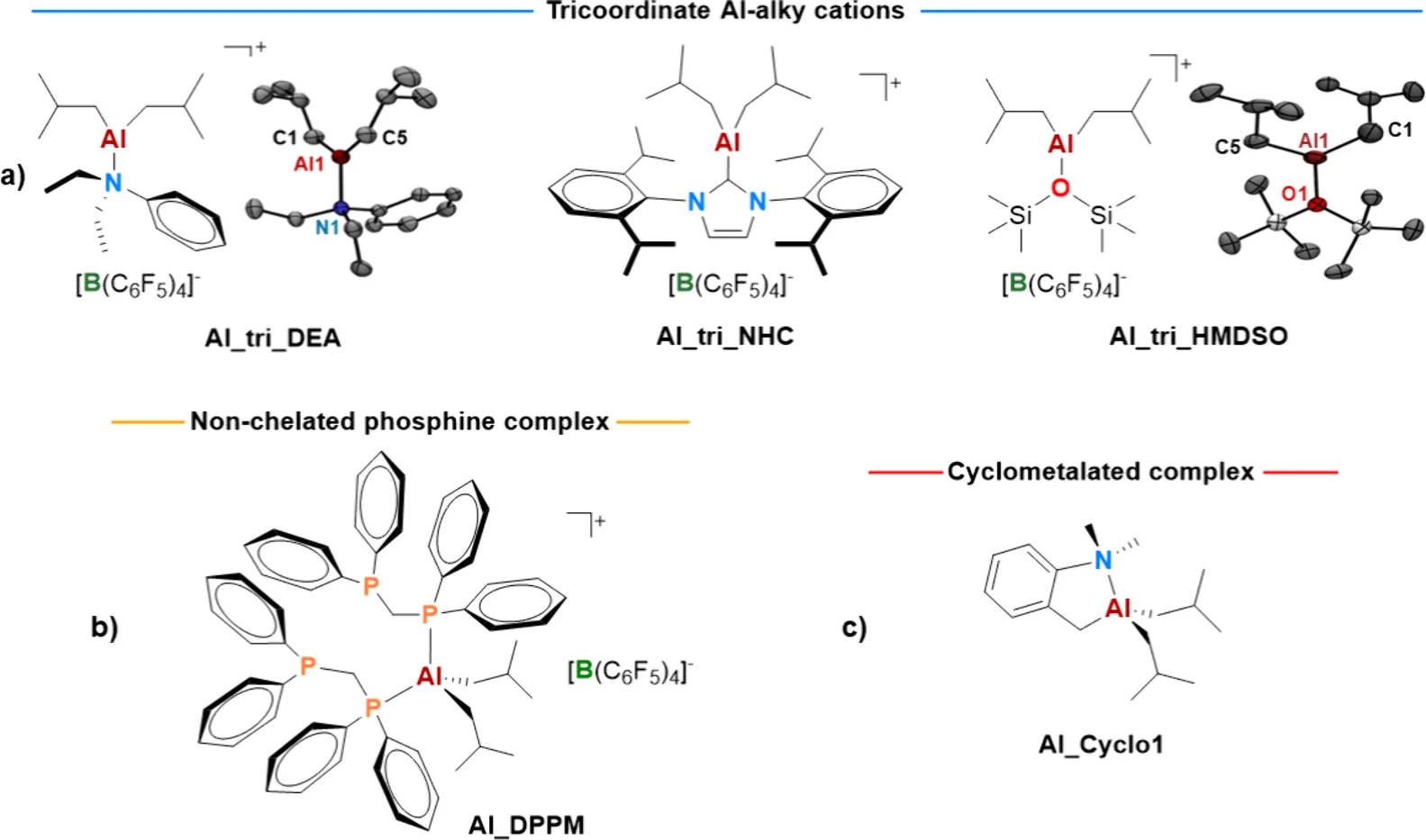
Unexpected yet stable products isolated from attempts to synthesize **AlHAl_L**, comprising (a) borate salts of tricoordinate Al-alkyl cations, (b) **Al_L** with two monocoordinated diphosphines, and (c) neutral cyclometalated species.

By evaluating the effects of **L** variation on the relative stability of mono- and dinuclear species based on the equilibrium:

1 $$\mathrm{Al}\_L+{\left[\mathrm{Al}i{\mathrm{Bu}}_{2}H\right]}_{3}⇄\mathrm{AlHAl}\_L+{\left[\mathrm{Al}i{\mathrm{Bu}}_{2}H\right]}_{2}$$

which accounts for the self-aggregation tendency of DIBAL-H,^1^ the ligands can be grouped in three classes:

(a) Anilines and amines, which favor the formation of dinuclear **AlHAl_L** (Δ*G* ≈ −1.5 kcal/mol; Table 2, entries 1–8).
(b) Monodentate aromatic phosphines, **DPPB** and **DPE**, which favor the formation of mononuclear **Al_L** (Δ*G* ≈ 1.5 kcal/mol; entries 11–13, 15).
(c) N-Heterocycles, **DPPE**, **DPPP**, and **THF**, which strongly favor the formation of mononuclear **Al_L**, leading to no detectable amounts of **AlHAl_L** (Δ*G* ≫ 0 kcal/mol; entries 9, 10, 14, 16).

**Table 2:** Equilibrium Constants (*K*_eq_) and Gibbs Free Energy Differences (Δ*G*) for the Reaction Reported in eq 1, and ^^1^^H NMR Chemical Shifts of Bridging Hydrides of **AlHAl_L** at 298 K (δ)

entry	L	solvent	*K*_eq_	Δ*G* (kcal/mol)	δ(^1^H) ppm
1	**DMA**	toluene-*d*_8_	12	–1.5	2.80
2	**DMA**	chlorobenzene-*d*_5_	12	–1.5	2.86
3	**DMA-C**_**16**_	toluene-*d*_8_	23	–1.9	2.91
4	**DMA-Ph**	toluene-*d*_8_	11	–1.4	2.89
5	**DMA-Cl**	chlorobenzene-*d*_5_	11	–1.4	2.86
6	**DMCA** ^a^	chlorobenzene-*d*_5_	>23	–1.9	2.87
7	**DMHA** ^b^	toluene-*d*_8_	13	–1.5	2.63
8	**N-heterocycles** ^a^	toluene-*d*_8_	≪1	≫0	na

9	**DMPP** ^a^	toluene-*d*_8_	≪1	≫0	na
10	**PPh**_**3**_	toluene-*d*_8_	0.07	1.6	3.60
11	**4-Me-PPh**_**3**_	toluene-*d*_8_	0.10	1.4	3.59
12	**4-F-PPh**_**3**_	chlorobenzene-*d*_5_	0.13	1.2	3.48
13	**DPPE** and **DPPP**	chlorobenzene-*d*_5_	≪1	≫0	na
14	**DPPB**	chlorobenzene-*d*_5_	0.06	1.7	3.28

15	**THF** ^a^	chlorobenzene-*d*_5_	≪1	≫0	na
16	**DPE**	chlorobenzene-*d*_5_	0.06	1.7	3.79

a No absolute values could be determined, as the concentration of some relevant species is below the detection limits of NMR.

b Limited Stability (see ref 2). Data from refs 2 and 3 .

A similar classification can be made based on the NMR chemical shift of the bridging hydride (Table 2).

The largest variations were therefore observed among the three classes rather than among the members of each class. Less hindering and more basic ligands like N-heterocycles appear to form more stable mononuclear **Al_L** compared to, *e.g.*, **L** = anilines. The same trend emerges between **DMA** and its P-analogue **DMPP**, which is more coordinatingthan the former and leads to less congested metal centers due to the longer Al–P vs Al–N bond.^3^ For bidentate ligands, the dominant factor appears to be the length of the bridge between the two P atoms, which is ideal for **DPPE** and **DPPP** for chelating around one Al center, while better suited for a bridging ligand with **DPPB**; it is worth mentioning that these species represent very rare examples of stable cationic Al-phosphine complexes.^79,102,103^

With the only exceptions of **Py-4-NMe**_**2**_ and **DPE**, all isolated AAB salts are effective cocatalysts, as demonstrated with **Cat-2** in ethene/1-hexene copolymerization.^2,3^ To enable a fair comparison, an equimolar mixture of **Al_L** or tricoordinate species (Figure 5a) and DIBAL-H was used irrespectively of the *K*_eq_ for eq 1. Variations in productivity (*R*_p_; Figure 6) are broad and do not correlate with the relative stability of mono- and dinuclear species. Marked electronic effects emerge among N-heterocycles and phosphine-derived AAB salts, with electron-withdrawing substituents leading to higher productivity (e.g., **Py-3,5-F** vs **Py-3,5-Me** and **4-F-PPh**_**3**_ vs **4-Me-PPh**_**3**_). Considering that decoordination of a ligand from saturated four-coordinate Al centers is likely necessary for reactivity (*vide infra*), these effects were tentatively ascribed to electron-withdrawing groups favoring precatalyst activation. The same effects are not observed among anilines, likely because decoordination is intrinsically easier with these less-coordinating ligands.

**Figure 6:**
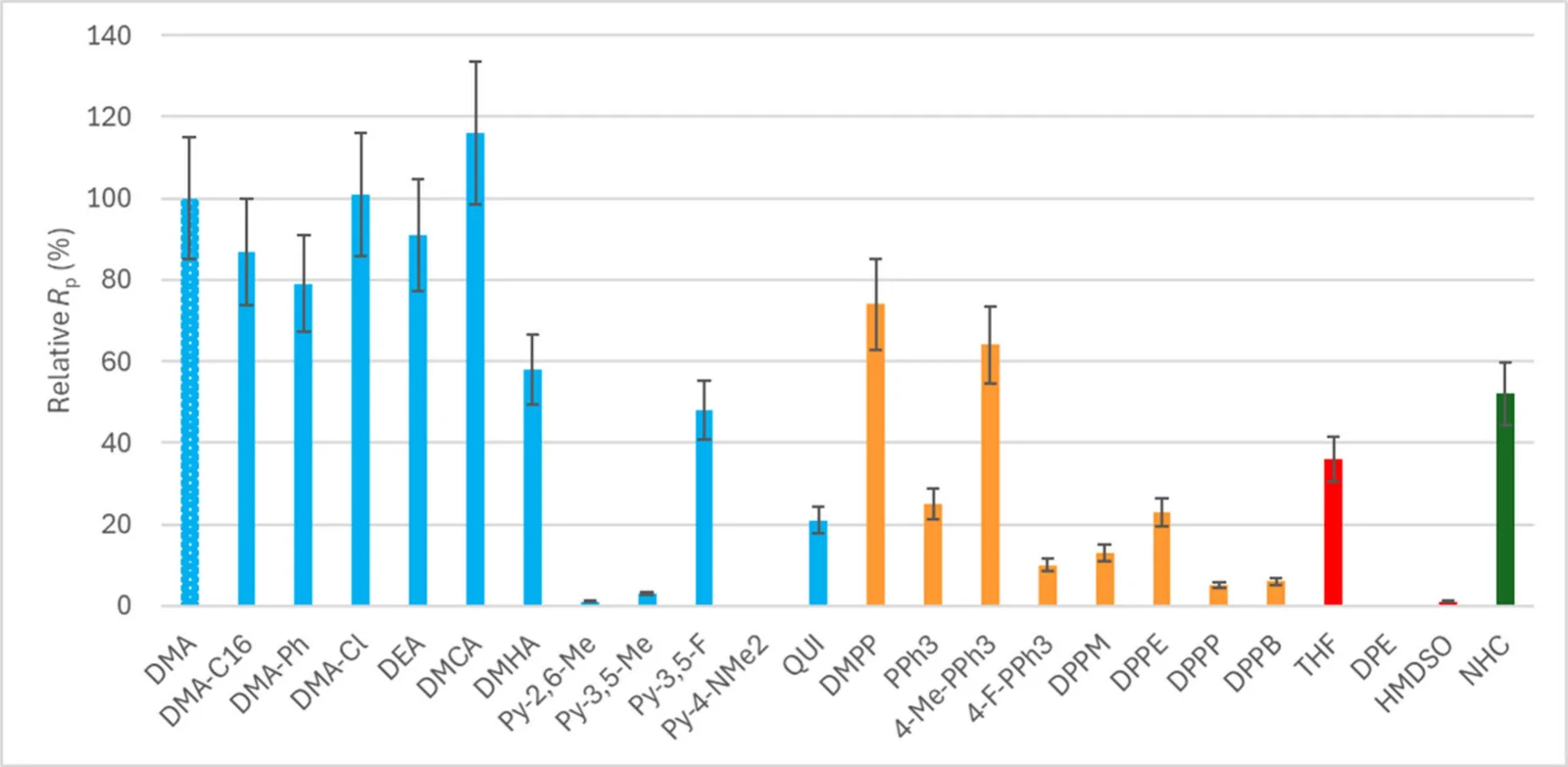
Relative productivities obtained with **Cat-2** in combination with the various AAB cocatalysts with respect to that with **AlHAl_DMA** (ethylene/1-hexene copolymerization, toluene, 60 °C, 4.5 bar, *C*_hexene_ = 1.8 v/v%, [Zr] = 3 × 10^–7^ M; [B] = 3 × 10^–5^ M). Data are averages of at least duplicates and are taken from refs 2, 3 .

Preliminary quantitative structure–properties relationships (QSPR) models on N-donor ligands support these hypotheses.^2^ Nevertheless, fully rationalizing these trends is difficult owing to the numerous subtleties determining productivity in olefin polymerization,^29,104–108^ and the many structural parameters potentially playing a role in determining stability and reactivity of AAB salts. Although a larger data set would be necessary to analyze these systems, for instance, with machine learning algorithms, the available set of data is already a testament to the potentiality of **AlHAl**-type activators in terms of property tuning.

The same polymer was obtained with nearly all cocatalysts, suggesting that the same active species were formed. Only bidentate phosphines and **4-F-PPh**_**3**_ led to appreciably lower comonomer incorporation, without affecting the polymer MW. This was tentatively traced to catalyst-cocatalyst interactions,^3^ similar to the above-mentioned case reported by Rieger and co-workers.^82^

Notably, beyond reactivity, also physicochemical properties of AAB salts can be tuned by structural variations. For instance, installing long alkyl substituents on **L** as in **AlHAl_DMA-C**_**16**_ improves solubility and enables polymerization in saturated hydrocarbon solvents (Figure 7),^101^ which are typically preferred to aromatics for industrial applications.^53,54,58,59^ Since ion pairing effects are strongly dependent on solvent polarity, this represents another important handle for tuning productivity and selectivity.^35,105,109,110^ For instance, Marks and co-workers recently reported that using a TTB analogue with long alkyl substituents enables ethylene polymerization by a constrained geometry catalysts in saturated hydrocarbons, where the catalyst selectivity shifts from producing linear to hyperbranched polyethylene: an unprecedented result for group 4 metal catalysts.^54^

**Figure 7:**
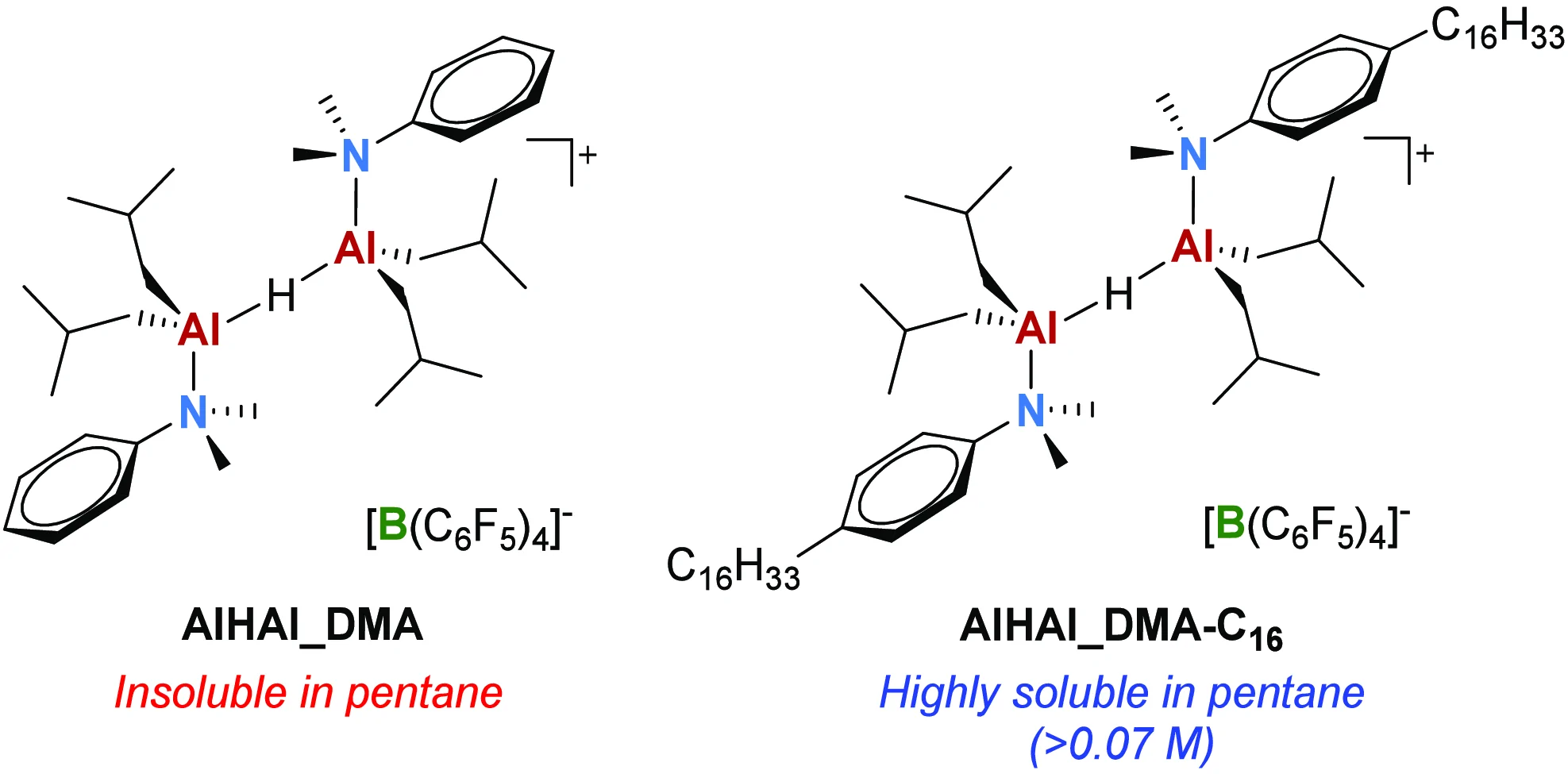
Comparison between **AlHAl_DMA** and its hydrocarbon soluble analogue **AlHAl_DMA-C**_**16**_.

Even though no new **AlHAl_L** significantly outperformed the **AlHAl_DMA** prototype (Figure 6), these studies have successfully demonstrated that a broad family of tunable AAB salts acting as MAO’s molecular cousins can be accessed. Cocatalyst tuning is not *per se* unprecedented, as numerous examples of cation or anion engineering of organic borate salts^12,29,52–57,111–113^ and modifications of MAO^43,114–122^ are known. Nevertheless, **AlHAl_L** variations offer significant advantages, since they are far simpler than, *e*.*g.*, engineering of perfluoroaromatic borate anions and, in difference to MAO, they can be easily studied at a molecular level. The results depicted herein should be therefore regarded as the tip of the iceberg of **AlHAl_L** potentialities. Although the prototypical **AlHAl_DMA** remains the best performing cocatalyst, it might be expected that other AAB salts will prove more effective in combination with different precatalysts and/or under different reaction conditions. Furthermore, activators that lead to somewhat lower productivities might be even preferable to control polymerization kinetics with superactive catalysts.^72^ This opens the way for a “mix-and-match” approach, possibly aided by machine learning, in which precatalyst and cocatalyst are optimized in combination and not in parallel as it has occurred traditionally.

## 5 Mechanism of Action of **AlHAl_DMA**

Polymerization experiments demonstrate that **AlHAl_DMA** and its homologues function as a complete cocatalyst but do not provide clear indications on how they fulfill this role. As discussed in the Introduction, activation of dichloride precatalysts consists of two steps, namely Cl-abstraction and Cl-exchange with a group capable of undergoing olefin migratory insertion (Scheme 1a,b). Regarding abstraction, the aluminum sites of **AlHAl_DMA** are coordinatively saturated and yet behave as strong Lewis acids capable of ionizing the precatalysts. The same characteristic is found in MAO and was described by Barron and co-workers in terms “latent Lewis acidity”.^123^ Following this analogy, it is reasonable to assume that **AlHAl_DMA** releases a [Al*i*Bu_2_(**DMA**)]^+^ transient cation acting as a chloride abstractor, similarly to AlMe_2_^+^ from MAO (Scheme 1a),^62–67^ and that the subsequent exchange of the second chloride involves the remaining Al*i*Bu_2_(**DMA**)­H subunit (Scheme 4).

**Scheme 4:**
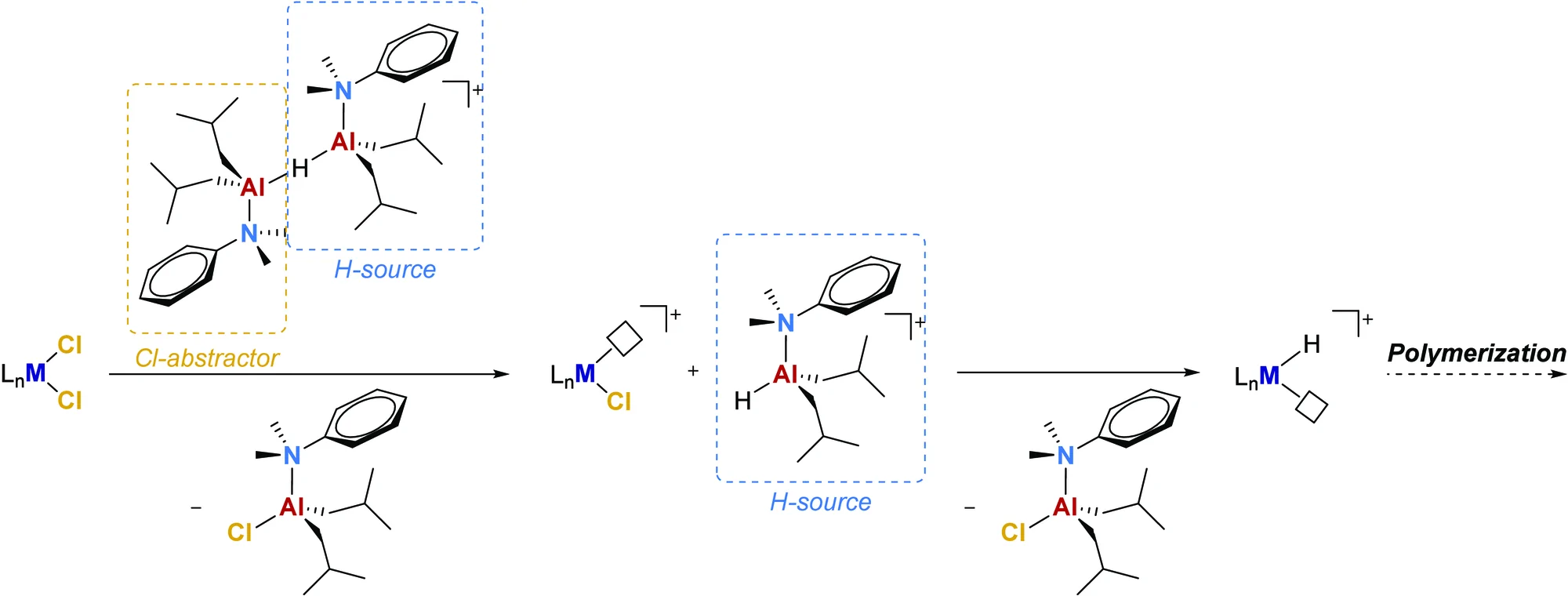
Proposed Precatalyst Activation Mechanism by **AlHAl_DMA** ^a^

a Adapted with permission from ref 4. Copyright 2025 American Chemical Society.

Strong supporting evidence for this mechanistic hypothesis has been reported.^4^ The key to investigate this reactivity step by step was using tricoordinate [Al*i*Bu_2_(**L**)]^+^ cations isolated with **DEA**, **HMDSO** and **NHC** (Figure 5a), serving as suitable models for the non-isolable [Al*i*Bu_2_(**DMA**)]^+^.^4^ Reaction with (Me_2_SiCp_2_)­ZrCl_2_ led to the clean formation of singly bridged Zr/Al heterodinuclear adducts **2_DEA** and **2_NHC** (Scheme 5a,b), which correspond to the expected intermediates for chloride abstraction by [Al*i*Bu_2_(**L**)]^+^. These are rather unique examples of cationic and singly bridged heterodinuclear species, as only neutral analogues had been reported previuosly.^123–129^

**Scheme 5:**
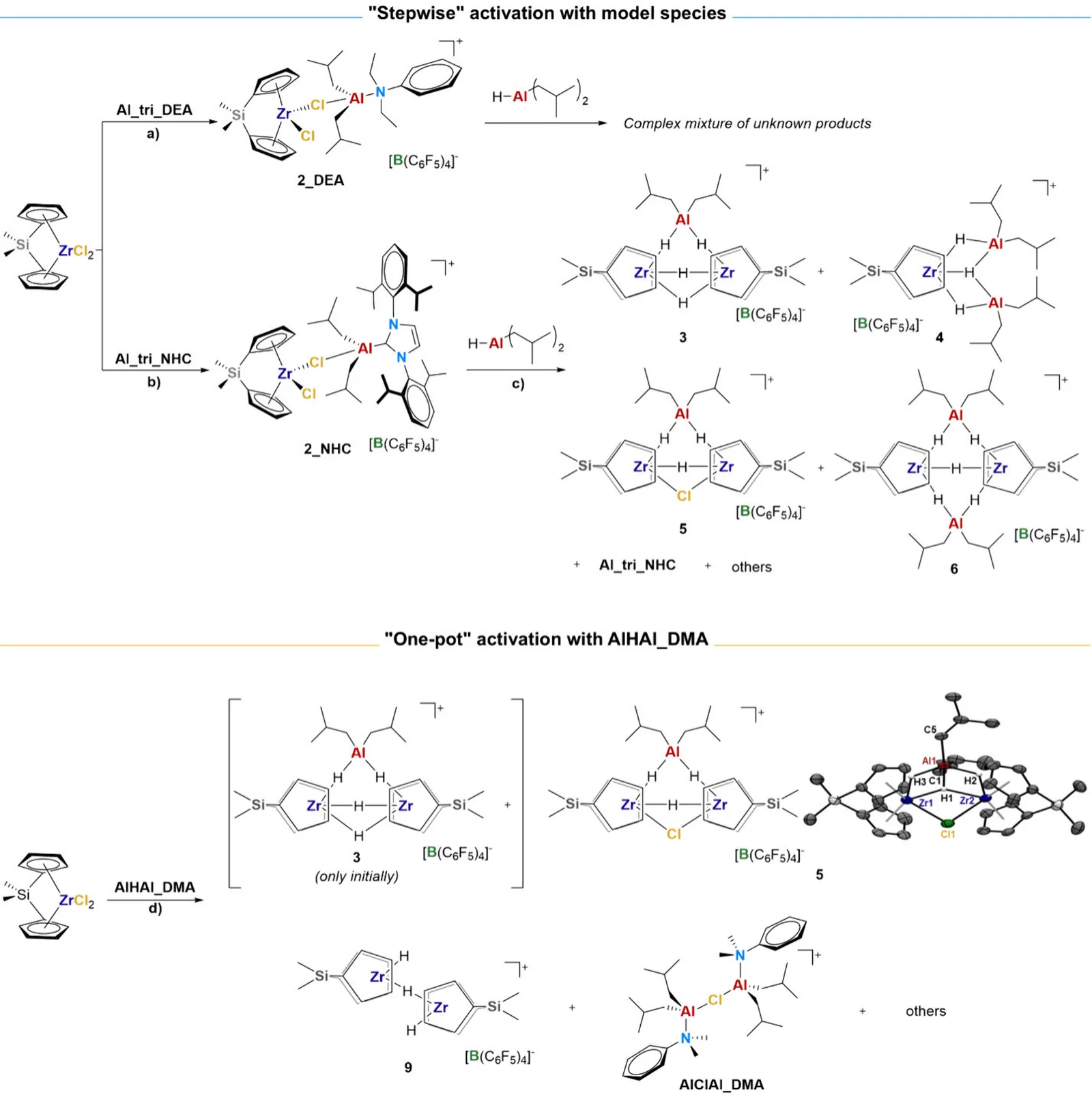
Activation of a Zirconocene Dichloride Using Tricoordinate Model Species (a,b) and DIBAL-H (c) or AlHAl_DMA (d), as Studied by NMR Spectroscopy

Reaction of these adducts with DIBAL-H was then used to model the subsequent Cl-exchange step. Like Al*i*Bu_2_(**DMA**)­H, DIBAL-H can serve as *iso*-butyl or hydride donor to the zirconium center, and they both represent suitable groups to initiate polymerization. In the case of **2_NHC**, the major products correspond to the multinuclear adducts **3**–**6** shown in Scheme 5c, some of them resembling previously reported species obtained with TTB or AB/TIBAL.^70,130–132^ Only μ-Cl and μ-H bridging moieties were observed, suggesting that Zr–H, not Zr–*i*Bu active species are preferentially formed.^4^

Some of these products, namely **3** and **5**, were also observed in the “one-pot” activation reaction between (Me_2_SiCp_2_)­ZrCl_2_ and **AlHAl_DMA**, consistently with their relevance in the activation chemistry of this cocatalyst. In this case, however, additional species form and become dominant at longer reaction times like the homodinuclear Zr-hydride adduct **9** (Scheme 5d). Chloride transfer from Zr to Al was evidenced by the detection of the chlorinated analogue of **AlHAl_DMA** (**AlClAl_DMA**; Scheme 5d).^4^

The latter species, featuring a bridging chloride rather than a hydride, could be also independently synthesized by the reaction of **Al_DMA** and di-*iso*-butyl aluminum chloride. **AlClAl_DMA** is unable to activate group 4 metal precatalysts, unless TIBAL is added, consistent with the hypothesis that the hydride is needed for precatalyst activation (Scheme 4).^1^

Interestingly, the hydride appears to be crucial also for the excellent scavening properties of **AlHAl_DMA**. NMR spectroscopic studies revealed that, upon exposure to air, the Al–(μ-H)–Al fragment rapidly disappears, while some Al–*i*Bu and Al–DMA groups remain visible since **Al_DMA** forms. Also other products form and become dominant over time at the expense of **Al_DMA** decomposition, namely *iso*-butane (from hydrolysis of Al–*i*Bu), *iso*-butene (from Al–*i*Bu via β-H elimination) and the Al-alkoxide species **12** (Scheme 6). Eventually, broad resonances likely corresponding to *iso*-butylalumoxane oligomers, are detected.^4^ This comprehensive mechanistic study highlights once more the advantage of well-defined AAB salts over the elusive MAO.

**Scheme 6:**
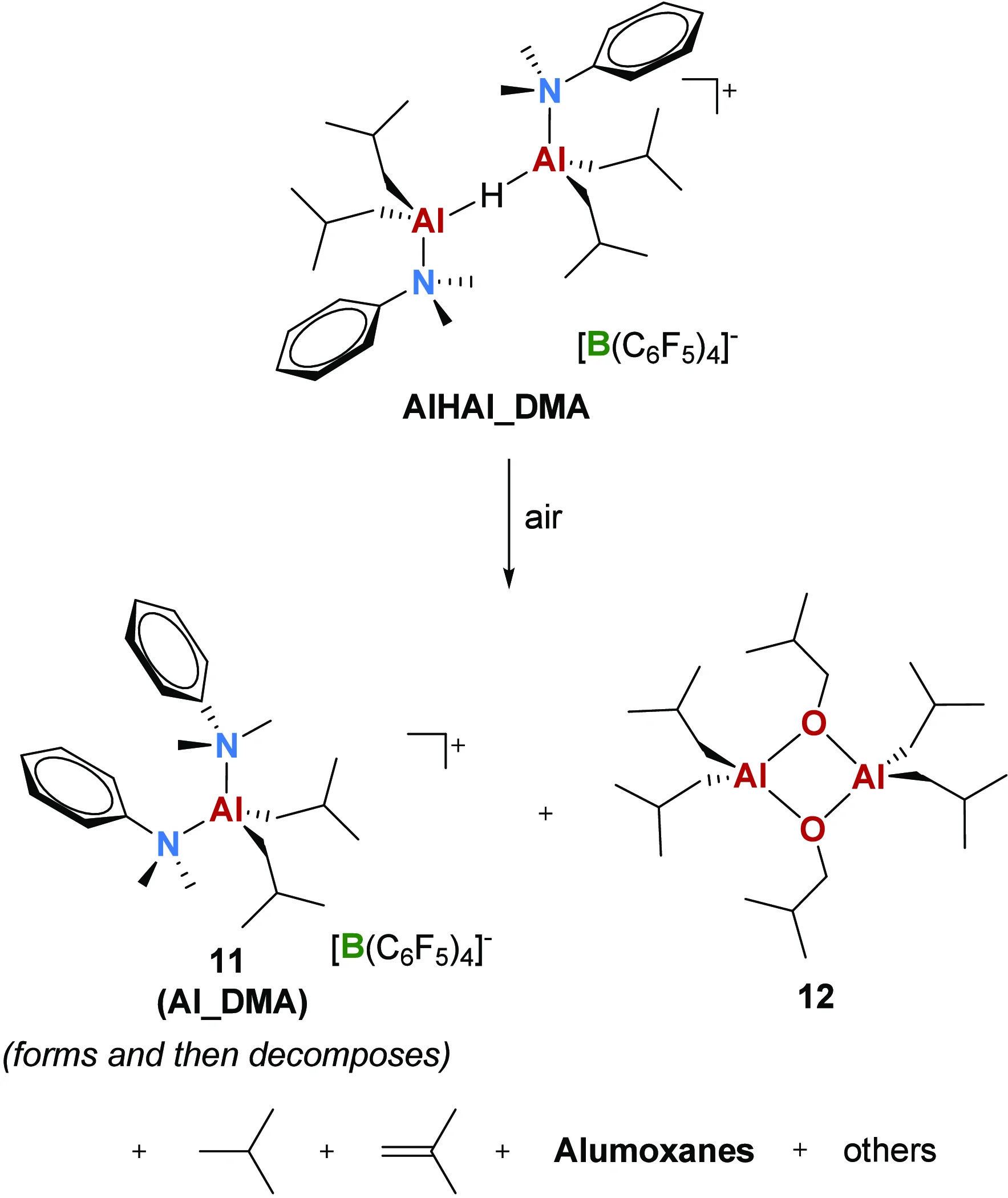
Reactivity of AlHAl_DMA upon Exposure to Air

## 6 Conclusions

The success story of molecular olefin polymerization is far from being over. Lately, the scene has been dominated by precatalyst development, while innovation in cocatalysts has largely stalled.

“MAO’s molecular cousins” hold the promise of a plot twist, as their combination of well-defined structures and complete reactivity overcomes the limitations of established cocatalysts. Their industrial application is arguably hampered by the high costs; furthermore, in difference to MAO,^133^ they can *not yet* be easily heterogenized on a support for applications in industrial gas-phase polymerization processes. Nevertheless, the possibility of tailoring their properties for specific precatalysts and processes, so far only preliminarily demonstrated, could be exploited to produce niche materials for high-end applications that are hardly accessible with conventional methods. To this end, the main challenge lies in exploring the hyperspace of variables controlling catalytic performance in polymerization, which is very broad by default and further expanded by the numerous tuning handles introduced by **AlHAl**-type cocatalyst. Rationalizing QSPR is key to truly exploit their protential and provide a clear pratical advantage with respect to established systems.

Nonetheless, beyond any doubt, “MAO’s molecular cousins” are relevantfrom a fundamental point of view. The reactivity of the established cocatalyst centers around its ability to generate highly reactive AlR_2_^+^ cations. **AlHAl_DMA** and its homologues demonstrate that such species can be tamed without killing their desirable reactivity, opening the way for mechanistic investigation and molecular design. Furthermore, the peculiar structure of these compounds may be relevant also in the broader context of organoaluminum chemistry and catalysis.

## Abbreviations

AAB: aluminum alkyl borate
MAO: methylaluminoxane
TIBAL: tri-iso-butylalluminium
DIBAL-H: di-iso-butylaluminum hydride
TTB: trityl borate
AB: anilinium borate
DMA: *N*,*N*-dimethylaniline
MW: molecular weight.
